# Association of Subjective Memory Complaints With White Matter Hyperintensities and Cognitive Decline Among Older Adults in Chicago, Illinois

**DOI:** 10.1001/jamanetworkopen.2022.7512

**Published:** 2022-04-15

**Authors:** Anisa Dhana, Charles DeCarli, Klodian Dhana, Pankaja Desai, Kristin Krueger, Denis A. Evans, Kumar B. Rajan

**Affiliations:** 1 Rush Institute for Healthy Aging, Rush University Medical Center, Chicago, Illinois; 2 Department of Internal Medicine, Rush University Medical Center, Chicago, Illinois; 3 Department of Neurology, University of California at Davis, Sacramento

## Abstract

**Importance:**

Subjective memory complaints (SMCs) are associated with a faster cognitive decline; whether this association is also associated with structural brain alterations, such as white matter hyperintensity (WMH) volumes, requires investigation.

**Objective:**

To evaluate the association of SMCs with WMH volumes and cognitive decline and investigate the role of WMH volumes in the association between SMCs and cognitive decline.

**Design, Setting, and Participants:**

The Chicago Health and Aging Project, a population-based cohort study, enrolled adults aged 65 years or older. Data collection occurred in 3-year cycles from 1993 until 2012. Our study comprised 975 participants with magnetic resonance imaging assessments, of which 900 participants had data on SMCs and covariates, and 713 participants provided 2 or more cognitive assessments during the follow-up. Statistical analyses were conducted from May to October 2021.

**Exposures:**

SMCs were obtained from self-reported questionnaire data during clinical evaluations, and the cycle, when reported, constituted the baseline of our study. Based on the frequency and severity of concerns, we categorized participants into 3 groups, (1) no concerns, (2) moderate concerns, and (3) very worried.

**Main Outcomes and Measures:**

Volumetric magnetic resonance imaging measures of WMH volume and neuropsychological testing assessments of global cognition. Linear regression analysis was used to investigate the association between SMCs and WMH volumes in a multivariable model adjusted for age, sex, race and ethnicity, education, *APOE4* status, and total intracranial volume. The association of SMCs with cognitive decline was investigated using linear mixed-effects models for age, sex, race and ethnicity, education, *APOE4* status, follow-up time, and each variable in interaction with time to estimate the annual longitudinal change in cognitive function.

**Results:**

Of the 900 participants with data on SMCs, covariates, and WMH volumes, 553 (61.4%) were women, 539 (59.9%) were African American, and the mean (SD) age was 79.5 (6.2) years. SMCs were associated with a larger WMH volume and faster cognitive decline. Compared with participants with no concerns, participants who were very worried had higher WMH volumes (β = 0.833; 95% CI, 0.203-1.463) and 174% faster cognitive decline (β = −0.049; 95% CI, −0.076 to −0.022). The association between SMCs and cognitive decline remained statistically significant among individuals with large WMH volumes (ie, within the fourth quartile). Within the fourth quartile of WMH volumes, participants who were very worried had 428% faster cognitive decline (β = −0.077; 95% CI, −0.144 to −0.011) compared with participants with no concerns.

**Conclusions and Relevance:**

This cohort study suggests that SMCs, frequently reported by older individuals, are an important sign of cognitive impairment, especially among people with abnormalities in brain structure, such as larger WMH volumes.

## Key Points

**Question:**

Are subjective memory complaints (SMCs) associated with cognitive decline because of structural brain alterations, such as white matter hyperintensity (WMH) volumes?

**Findings:**

In a population-based cohort study of 900 participants with data on SMCs, covariates, and WMH volumes, SMCs were associated with larger WMH volumes and faster cognitive decline. The association of SMCs with cognitive decline was significant among individuals with higher WMH volumes.

**Meaning:**

This study suggests that SMCs are an important sign of cognitive impairment, especially for individuals with a higher WMH volume.

## Teaser

This cohort study evaluates the association of subjective memory complaints with white matter hyperintensity volumes and cognitive decline and investigates the role of white matter hyperintensity volume in the association between subjective memory complaints and cognitive decline in a biracial, population-based cohort study of older adults in Chicago, Illinois.

## Introduction

Alzheimer dementia, a progressive brain disorder that impairs memory, thinking, and decision-making abilities, is a leading cause of disability in a growing aging population.^1,2^ While it is a devastating disease for our society, Alzheimer dementia has a long prodromal phase, providing a critical time window for potential interventions to successfully postpone or prevent the disease’s onset.^3,4^ Therefore, it is a public health priority to identify people at very early stages of cognitive impairment and consequently to prevent or postpone neurodegenerative diseases.^5^

In recent years, a growing body of research has shown that individuals with no evident objective cognitive impairment but who demonstrate concerns about everyday memory, known as subjective memory complaints (SMCs), are at high risk of faster cognitive decline and developing incident dementia.^6,7,8^ SMCs have been suggested as an early sign of cognitive impairment.^8,9^ Several studies have investigated whether SMCs result from differences in brain structure, specifically white matter hyperintensities (WMHs),^10^ but the data are limited in diverse communities, including among African American individuals. The alterations in brain structure, such as larger WMH volumes in elderly individuals, are potentially irreversible,^11^ questioning the effect of primary prevention of cognitive deterioration and dementia in people living with SMCs. Therefore, determining whether SMCs are in part a consequence of brain structure abnormalities, such as WMHs, is an essential step toward understanding the origin of SMCs and ultimately developing tailored preventive strategies for these high-risk populations. In addition, the prevalence of vascular risk factors strongly associated with WMHs differs by communities with the highest prevalence in African American communities,^12^ suggesting that the association of SMCs with WMHs and cognitive impairment may vary by race and ethnicity.

Our study aims are (1) to evalute the association of SMCs with WMH volumes and cognitive decline and (2) to investigate the role of WMH volume in the association between SMCs and cognitive decline in a biracial, population-based cohort study of older adults from a geographically defined community in Chicago, Illinois.

## Methods

### Study Design, Settings, and Population

The Chicago Health and Aging Project is a longitudinal population-based study investigating risk factors for cognitive impairment and Alzheimer disease and related dementias.^13^ Participants (n = 10 802), African American individuals and White individuads, both older than 65 years, were recruited through a door-to-door census. Data collection occurred in 3-year cycles from 1993 until 2012. Of 10 802 individuals included in the study, 2932 (27.1%) were randomly selected for clinical evaluations of prevalent and incident dementia. Of these participants with clinical evaluations, 1235 were invited for magnetic resonance imaging (MRI) scans from 2000 to 2012, and 975 consented to participate in the MRI study. Participants’ demographic characteristics (eg, sex and race and ethnicity) and baseline cognitive functioning (*P* = .28) did not differ between the population and the MRI samples. Of the 975 participants with MRI data, 900 had data on SMCs and covariates, and 713 provided 2 or more cognitive assessments during the follow-up. The mean (SD) number of cognitive assessments was 3.1 (1.0) during the follow-up, each approximately 3 years apart.

The Chicago Health and Aging Project study was approved by the Rush University Medical Center institutional review board, and all participants provided written informed consent. This study followed the Strengthening the Reporting of Observational Studies in Epidemiology (STROBE) reporting guideline for observational studies.

### Assessment of SMCs

In the Chicago Health and Aging Project, participants were asked 2 questions about their subjective cognitive or memory complaints: (1) “How often do you have trouble remembering things?” (2) “Compared with 10 years ago, would you say that your memory is?” The answers were given based on a 5-point Likert scale ranging from 1 (poor) to 5 (better). Based on a previous publication,^6^ we summed the 2 memory scores and calculated each individual’s overall memory complaint score, ranging from 2 to 10. Next, we grouped participants into 3 categories based on frequency and severity of concerns; the first group included participants with no concerns who had scores ranging from 7 to 10, the second group included participants with moderate concerns who had scores ranging from 4 to 6; and the third group included participants who were very worried and had scores ranging from 2 to 3.

### Assessment of Demographic Characteristics and Other Confounders

Age was computed from the self-reported birth date and the dates of SMCs and MRI assessments. Race and ethnicity and sex were self-reported using structured questions based on the 1990 US Census. The study participants had no a priori exclusions but were from a biracial population. Education was based on self-reported years of regular schooling. The *APOE* genotypes were determined based on the single-nucleotide variations of rs7412 and rs429358 measured by the Broad Institute Center for Genotyping using the hME Sequenom MassARRAY platform.^14^ Information on comorbid conditions (including stroke, cancer, diabetes, hypertension, heart disease, and hip fracture) was acquired by self-report questions from the Established Populations for Epidemiologic Studies of the Elderly.^15^

### Assessment of WMH

From 2000 to 2012, participants with a clinical evaluation for Alzheimer disease (one-third of the study participants) were invited to participate in the MRI study. WMH is assessed on a combination of fluid-attenuated inversion recovery and 3-dimensional T1 MRI scans using a modified bayesian probability structure based on a previously published method of histogram fitting.^16,17^ These MRI scans are for research purposes. Prior probability maps for WMH were created from more than 700 individuals with semiautomatic detection of WMH followed by manual editing.^16^ Likelihood estimates of the native image are calculated through histogram segmentation and thresholding.^16^ All segmentation is initially performed in standard space resulting in probability likelihood values of WMH at each voxel in the white matter. These probabilities are thresholded at 3.5 SD above the mean to create a binary WMH mask.

Further segmentation is based on a modified bayesian approach that combines image likelihood estimates, spatial priors, and tissue class constraints.^17^ The segmented WMH masks are then back-transformed to native space for tissue volume calculation. Volumes are log-transformed to normalize variance.

### Assessment of Global Cognitive Function

Four neuropsychological tests of cognitive function were conducted during in-home interviews in each Chicago Health and Aging Project cycle: immediate and delayed recall of a brief story^18,19^; the Symbol Digit Modalities Test^20^; a measure of perceptual speed^21^; and the Mini-Mental State Examination.^22^ Each test was transformed from raw score to *z* score using the mean (SD) baseline population scores. After that, we created a composite *z* score by averaging all 4 tests. A positive *z* score indicates better cognitive performance than the mean population score, and a negative score indicates a poor cognitive score. Global cognition was computed on each cycle for all study participants, and based on the cognitive performance (eg, scoring) during the follow-up, we determined the rate of cognitive decline.

### Statistical Analysis

We used mean values, geometric mean values, and SDs for continuous variables and absolute numbers and percentages for categorical variables to describe the characteristics of the study participants. Linear regression analysis was used to investigate the association between SMCs and WMH volumes in a multivariable model adjusted for age, sex, race and ethnicity, education, *APOE4* status, and total intracranial volume. The association of SMCs with cognitive decline was investigated using linear mixed-effects models for age, sex, race and ethnicity, education, *APOE4* status, follow-up time, and each variable is in interaction with time to estimate the annual longitudinal change in cognitive function.

We evaluated the role of WMH volume in the association between SMCs and cognitive decline by examining this association across different levels of WMH volumes. In this analysis, we standardized the WMH volume to total cranial volume and created quartiles to categorize study participants by the level of WMH volumes. Then, we evaluated the association between SMCs and cognitive decline in each quartile of WMH. In additon, we developed a multivariable-adjusted model including WMH volumes and evaluated the significance of the association between SMCs and cognitive decline.

As a sensitivity analysis, we considered (1) whether age, sex, and race and ethncity modified the association of SMCs with WMH volumes or the association of SMCs with cognitive decline; (2) whether baseline cognitive function had a role in the association between SMCs and WMH volume; and (3) additional adjustment by comorbid conditions (including stroke, cancer, diabetes, hypertension, heart disease, and hip fracture) in the multivariable model evaluating the association of SMCs with WMH volume and cognitive decline.

Analyses were conducted using R program, version 3.6 (R Group for Statistical Computing). Our a priori cutoff for statistical significance included *P* < .05. Hypothesis tests were 2-sided.

## Results

Table 1 shows the baseline characteristics of the overall study population stratified by the levels of SMCs. Of the 900 participants with data on SMCs, covariates, and WMH volumes, 553 (61.4%) were women, 539 (59.9%) were African American, and the mean (SD) age was 79.5 (6.2) years. Compared with participants with no concerns, participants who were very worried were slightly older (mean [SD] age, 78.8 [6.5] years vs 80.2 [6.0] years), and most were men (62 of 158 [39.2%] with no concerns vs 38 of 84 [45.2%] who were very worried) and African American (86 of 158 [54.4%] with no concerns vs 57 of 84 [67.9%] who were very worried). Also, the mean (SD) number of years of education was 12.3 (3.5) for participants who were very worried and 13.2 (3.4) for participants with no concerns.

**Table 1.** Characteristics of the Study Population by Subjective Memory Complaints^a^

Characteristic	Participants, No. (%)			
	Overall (n = 900)	Subjective memory complaints		
		No concerns (n = 158)	Moderate concerns (n = 658)	Very worried (n = 84)
Age, mean (SD), y	79.5 (6.2)	78.8 (6.5)	79.6 (6.2)	80.2 (6.0)
Sex				
Female	553 (61.4)	96 (60.8)	411 (62.5)	46 (54.8)
Male	347 (38.6)	62 (39.2)	247 (37.5)	38 (45.2)
Race and ethnicity				
African American	539 (59.9)	86 (54.4)	396 (60.2)	57 (67.9)
Non-Hispanic White	361 (40.1)	72 (45.6)	262 (39.8)	27 (32.1)
Education, mean (SD), y	13.1 (3.5)	13.2 (3.4)	13.1 (3.5)	12.3 (3.5)
*APOE4* allele				
Noncarrier	604 (67.1)	112 (70.9)	441 (67.0)	51 (60.7)
Carrier	296 (32.9)	46 (29.1)	217 (33.0)	33 (39.3)
Chronic medical conditions^b^				
0	193 (21.4)	34 (21.5)	145 (22.1)	14 (16.6)
1	326 (36.2)	54 (34.2)	247 (37.5)	25 (29.8)
2	261 (29.1)	45 (28.5)	185 (28.1)	31 (36.9)
3-6	120 (13.3)	25 (15.8)	81 (12.3)	14 (16.7)
Global cognition, mean (SD), *z* score	0.28 (0.59)	0.34 (0.62)	0.28 (0.56)	0.17 (0.75)
White matter hyperintensities, geometric mean (SD) volume	6.8 (3.9)	5.93 (3.81)	6.78 (3.98)	8.59 (3.64)

Abbreviation: *APOE4*, apolipoprotein e4.

^a^ Composed of 3 groups: participants with no concerns included those who had no memory complaints, participants with moderate concerns included those with mild memory complaints, and participants who were very worried included those with substantial memory concerns. Details of memory complaints scoring are available in the Methods.

^b^ Chronic medical conditions include stroke, heart disease, cancer, diabetes, hypertension, and hip fracture.

Table 2 shows the association of SMCs categories with WMH volumes. Very worried participants had larger WMH volumes than their counterparts with no concerns (β = 0.833; 95% CI, 0.203-1.463; *P* = .01). Compared with participants with no concerns, very worried had higher of log-transformed WMH volumes. We detected no significant association with WMH volumes when comparing study participants with moderate concerns with participants with no concerns (β = 0.262; 95% CI, −0.134 to 0.657; *P* = .20).

**Table 2.** Association of Subjective Memory Complaints With White Matter Hyperintensity Volume and Global Cognitive Decline^a^

Subjective memory complaint	White matter hyperintensity volume		Global cognitive decline		
	β Estimate (95% CI)^b^	*P* value	β Estimate (95% CI)^c^	% of Change	*P* value
No concerns	0 [Reference]	NA	0 [Reference]	NA	NA
Moderate concerns	0.262 (−0.134 to 0.657)	.20	−0.014 (−0.033 to 0.005)	48.7 faster	.16
Very worried	0.833 (0.203 to 1.463)	.01	−0.049 (−0.076 to −0.022)	174 faster	<.001

Abbreviation: NA, not applicable.

^a^ Composed of 3 groups: participants with no concerns included those who had no memory complaints, participants with moderate concerns included those with mild memory complaints, and participants who were very worried included those with substantial memory concerns. Details of memory complaints scoring are available in the Methods.

^b^ Model is adjusted for age, sex, race and ethnicity, education, apolipoprotein e4, and total intracranial volume.

^c^ Model is adjusted for age, sex, race and ethnicity, education, apolipoprotein e4, and each of their interactions with time.

The association between SMCs and cognitive decline is also presented in Table 2. SMCs were associated with a faster rate of annual cognitive decline. Compared with individuals with no concerns, individuals who were very worried had an annual rate of cognitive decline of 0.049 units (β = −0.049; 95% CI, −0.076 to −0.022), corresponding to a 174% faster decline. There was no significant association with the rate of cognitive decline when we compared individuals with moderate concerns with individuals with no concerns (β = −0.014; 95% CI, −0.033 to 0.005; *P* = .16).

Table 3 presents the association of SMCs with cognitive decline across the levels of WMH volumes in the study sample because the participants were categorized into quartiles of WMH volumes standardized to total cranial volume. The association between SMCs and cognitive decline was statistically significant for participants with larger WMH volumes (eg, those within the the fourth quartile) but not for participants in the other groups (eg, those within the first to third quartiles). Among participants in the highest quartile of WMH, those who were very worried had an annual rate of cognitive decline of 0.077 units per year (β = −0.077; 95% CI, −0.144 to −0.011) that correspond to 427.8% faster decline compared with participants with no concerns (Table 3). The association between SMCs and cognitive decline was slightly attenuated when additionally adjusted by WMH in a multivariable-adjusted model. Compared with participants with no concerns, participants who were very worried had an annual cognitive decline of 0.047 units per year (β = −0.047; 95% CI, −0.074 to −0.021).

**Table 3.** Association of Subjective Memory Complaints With Global Cognitive Decline in Each Quartile of White Matter Hyperintensity Volume^a^

Subjective memory complaint^b^	White mater hyperintensity volume												
	First quartile (n = 181)			Second quartile (n = 179)			Third quartile (n = 177)			Fourth quartile (n = 176)		
	β (95% CI)^c^	% of Change	*P* value	β (95%CI)^c^	% of Change	*P* value	β (95%CI)^c^	% of Change	*P* value	β (95%CI)^c^	% of Change	*P* value
No concerns	0 [Reference]	NA	NA	0 [Reference]	NA	NA	0 [Reference]	NA	NA	0 [Reference]	NA	NA
Moderate concerns	0.011 (−0.029 to 0.051)	33.3 Slower	.60	−0.030 (−0.065 to 0.006)	93.8 Faster	.10	−0.008 (−0.042 to 0.026)	25 Faster	.65	−0.018 (−0.072 to 0.035)	100 Faster	.50
Very worried	0.00 (−0.054 to 0.055)	0 Slower	.99	−0.040 (−0.097 to 0.017)	125 Faster	.16	−0.045 (−0.098 to 0.008)	140.6 Faster	.09	−0.077 (−0.144 to −0.011)	427.8 Faster	.02

Abbreviation: NA, not applicable.

^a^ White matter hyperintensity volumes are standardized to total cranial volume.

^b^ Composed of 3 groups: participants with no concerns included those who had no memory complaints, participants with moderate concerns included those with mild memory complaints, and participants who were very worried included those with substantial memory concerns. Details of memory complaints scoring are available in the Methods.

^c^ Model is adjusted for age, sex, race and ethnicity, education, apolipoprotein e4, and each of their interactions with time.

No evidence was found that age, sex, or race and ethnicity modifies the association between SMCs and WMH or the association between SMCs and cognitive decline. No effect modification was found for the interaction term of SMCs with cognitive function status at baseline concerning WMH volumes. Additional adjustment by comorbid conditions did not change the strength or magnitude of the association between SMCs and WMH volumes or the association between SMCs and cognitive decline. Participants who were very worried had greater WMH volumes (β = 0.848; 95% CI, 0.218-1.477) and faster cognitive decline (β = −0.049; 95% CI, −0.076 to −0.021) than participants with no concerns (Table 4).

**Table 4.** Association of Subjective Memory Complaints With White Matter Hyperintensity Volume and Cognitive Decline in Model Adjusted for Chronic Medical Conditions^a^

Subjective memory complaint^b^	White matter hyperintensity volume		Global cognitive decline	
	β (95% CI)^c^	*P* value	β (95% CI)^d^	*P* value
No concerns	0 [Reference]	NA	0 [Reference]	NA
Moderate concerns	0.283 (−0.102 to 0.668)	.15	−0.014 (−0.033 to 0.005)	.16
Very worried	0.848 (0.218 to 1.477)	.008	−0.049 (−0.076 to −0.021)	<.001

Abbreviation: NA, not applicable.

^a^ Chronic medical conditions include stroke, heart disease, diabetes, hypertension, and hip fracture.

^b^ Composed of 3 groups: participants with no concerns included those who had no memory complaints, participants with moderate concerns included those with mild memory complaints, and participants who were very worried included those with substantial memory concerns. Details of memory complaints scoring are available in the Methods.

^c^ Model is adjusted for age, sex, race and ethnicity, education, apolipoprotein e4, and total intracranial volume.

^d^ Model is adjusted for age, sex, race and ethnicity, education, apolipoprotein e4, and each of their interactions with time.

## Discussion

In this biracial population-based study, SMCs were associated with larger WMH volumes and a faster cognitive decline. The association between SMCs, WMH volumes, and cognitive decline did not differ by age, sex, or race and ethnicity. The strength and magnitude of association between SMCs and cognitive decline were dependent on the levels of WMH volumes, with a significant association for participants with large WMH volumes and with a nonsignificant association for those with lower WMH volumes. These findings may suggest that SMCs could originate from different neural mechanisms, but when they coexist with larger WMH volumes, SMCs are an important sign that may indicate cognitive impairment in older adults.

Several epidemiological studies have investigated the association between SMCs and cognitive decline in elderly individuals.^6,23,24,25^ Results from these studies have shown that individuals who report concerns about their memory are at a higher risk of developing dementia than individuals who do not report memory complaints.^26,27^ For example, a large meta-analysis^23^ pooled results from 28 studies that included 29 723 individuals with a mean age of 71.6 years. Of the 29 723 individuals analyzed, 14 714 reported SMCs; thus, this meta-analysis^23^ concluded that older individuals with SMCs are twice as likely to develop dementia than people without SMCs. The studies included in this meta-analysis^23^ were primarily focused on community-based or memory clinics, which are prone to selection bias. In addition, the studies included in this meta-analysis^23^ did not investigate whether SMCs were associated with cognitive function or dementia differentially in African American individuals. In our investigation, we used data from a population-based study, which is advantageous for representing a general population and avoiding selection bias. Moreover, our study population included mostly African American individuals (ie, 539 of 900 [59.9%] were African American), allowing us to evaluate the association of SMCs across races and ethnicities. We found that SMCs were associated with a faster cognitive decline independent of race and ethncity.

While most of the research has been focused on SMCs and cognitive performance, studies investigating the associations between SMCs and structural brain alterations, such as WMH volumes, are limited.^28,29,30^ One of the studies investigating brain structure changes in association with subjective cognitive decline examined 67 healthy, late middle-aged and older adults for changes in WMH volumes.^30^ The authors reported that subjective cognitive decline was associated with larger WMH volumes.^30^ Our findings align with these results (ie, SMCs are associated with WMH volume). In addition, we provided novel evidence concerning the association between SMCs and cognitive decline among individuals with different WMH volumes. Specifically, we showed that the association between SMCs and cognitive decline is statistically relevant only among individuals with large WMH volumes. In addition, we provided novel evidence concerning the association between SMCs and cognitive decline among individuals with different WMH volumes. The fact that the association between SMCs and cognitive decline was significant among individuals with large WMH volumes might suggest 2 distinct mechanisms. First, it could be that SMCs are a symptom of vascular disease,^31^ and owing to greater WMH, these individuals experience a faster cognitive decline as they age. Second, it is also possible that SMCs and WMH share similar risk factors (eg, smoking and physical inactivity) and are developed independently as people age. When both are present in an individual, they may have a synergistic effect that significantly increases the risk of cognitive decline. We believe that the second mechanism is more likely in our study because when we adjusted for WMH volumes in a multivariable model, the estimates for the association between SMCs and cognitive decline did not change, suggesting an independent association. Nevertheless, although we investigated the association between SMCs and WMH volumes, we must acknowledge that other types of irreversible brain structural abnormalities (eg, hippocampal volume) could be important determinants for the presence of SMCs and shall be investigated in future studies.

### Limitations and Strengths

We acknowledge several limitations of our study. We had only 1 measurement of SMCs, and we do not know how long participants had these concerns. Knowing the years lived with SMCs would enable us to evaluate how fast those concerns developed and whether SMCs were present before participants developed larger WMH volumes. Because SMCs are self-reported by nature, they are prone to bias due to cognitive status at baseline; however, we examined the interaction term with cognitive function status at baseline and found no effect modification in association with WMH volumes. Participants who agreed to undergo an MRI assessment had no medical contraindications (eg, no heart pacemaker) at the time of the study, and therefore, our results may not be applicable to all individuals. The strength of this study includes the population-based study design, with the participation of African American individuals and non-Hispanic White individuals, and the availability of follow-up data to determine the cognitive changes as the study participants aged. The population-based cohort study is less likely to bias the results than a study that recruits participants from memory clinics.^32,33,34,35^ SMCs were assessed at the time when the participants underwent MRI, which allowed us to evaluate the association between SMCs and cognitive decline according to WMH volumes.

## Conclusions

In conclusion, this cohort study suggests that SMCs, which are frequently reported by elderly individuals, are an important sign of cognitive impairment, especially among individuals with abnormalities in brain structure, such as large WMH volumes. Obtaining information about SMCs from older adults may provide essential information about their future risk of cognitive impairment.
